# Bibliography

**Published:** 1900-07

**Authors:** 


### Bibliography.

TRANSACTIONS OF THE NATIONAL DENTAL ASSOCIATION. Including Proceedings of the Third Annual Session, held at Niagara Falls, N. Y., August, 1899; Proceedings of the Second Annual Session of the Southern Dental Association, Branch of the National Dental Association, held at New Orleans, La., February 9, 1899. S. S. White Dental Manufacturing Company, Philadelphia.

This volume exceeds in size any of the publications of the National Dental Association. This is partly due to combining the work of the Southern Branch with the proceedings of the main body, the first taking up two hundred and thirty-two pages and the latter four hundred and eighty-five, or seven hundred and seventeen in all. While this is the most voluminous report issued from this organization, it has the merit of being, probably, the best. Valuable papers are here given a permanent place in dental history. It would be difficult and invidious to select special papers for notice where all have a distinct value, but it is thought that Dr. Brophy's "Cure of Congenital Cleft Palate," while previously reported, must take rank as the most important contribution to surgery. Those who were present and witnessed the wonderful results obtained must give him credit for originating an operation more effective than anything heretofore attempted.

Space will not permit an extended notice of this valuable report. It is a striking illustration of the good to be accomplished through national bodies properly conducted. While the writer's ideal has not been reached in this direction, the fact remains that those who neglect the mingling with these associations gradually fail to remain in touch with the best thoughts in dentistry.

The S. S. White Dental Manufacturing Company are to be congratulated, not only in bringing out this report at an early date, but also in its careful and tasteful production.

